# Therapeutic Effects of rAAV-Mediated Concomittant Gene Transfer and Overexpression of TGF-β and IGF-I on the Chondrogenesis of Human Bone-Marrow-Derived Mesenchymal Stem Cells

**DOI:** 10.3390/ijms20102591

**Published:** 2019-05-27

**Authors:** Stephanie Morscheid, Ana Rey-Rico, Gertrud Schmitt, Henning Madry, Magali Cucchiarini, Jagadeesh Kumar Venkatesan

**Affiliations:** 1 Center of Experimental Orthopaedics, Saarland University Medical Center and Saarland University, D-66421 Homburg/Saar, Germany; StephiMeyer@web.de (S.M.); ana.rey.rico@gmail.com (A.R.-R.); gertrud.schmitt@uniklinikum-saarland.de (G.S.); henning.madry@uks.eu (H.M.); mmcucchiarini@hotmail.com (M.C.); 2 Department of Orthopaedic Surgery, Saarland University Medical Center and Saarland University, D-66421 Homburg/Saar, Germany

**Keywords:** cartilage repair, MSCs, rAAV vectors, TGF-β, IGF-I

## Abstract

Application of chondroreparative gene vectors in cartilage defects is a powerful approach to directly stimulate the regenerative activities of bone-marrow-derived mesenchymal stem cells (MSCs) that repopulate such lesions. Here, we investigated the ability of combined recombinant adeno-associated virus (rAAV) vector-mediated delivery of the potent transforming growth factor beta (TGF-β) and insulin-like growth factor I (IGF-I) to enhance the processes of chondrogenic differentiation in human MSCs (hMSCs) relative to individual candidate treatments and to reporter (*lacZ*) gene condition. The rAAV-hTGF-β and rAAV-hIGF-I vectors were simultaneously provided to hMSC aggregate cultures (TGF-β/IGF-I condition) in chondrogenic medium over time (21 days) versus TGF-β/*lacZ*, IGF-I/*lacZ*, and *lacZ* treatments at equivalent vector doses. The cultures were then processed to monitor transgene (co)-overexpression, the levels of biological activities in the cells (cell proliferation, matrix synthesis), and the development of a chondrogenic versus osteogenic/hypertrophic phenotype. Effective, durable co-overexpression of TGF-β with IGF-I via rAAV enhanced the proliferative, anabolic, and chondrogenic activities in hMSCs versus *lacZ* treatment and reached levels that were higher than those achieved upon single candidate gene transfer, while osteogenic/hypertrophic differentiation was delayed over the period of time evaluated. These findings demonstrate the potential of manipulating multiple therapeutic rAAV vectors as a tool to directly target bone-marrow-derived MSCs in sites of focal cartilage defects and to locally enhance the endogenous processes of cartilage repair.

## 1. Introduction

Articular cartilage lesions are prevalent clinical issues that impede the whole musculoskeletal system and for which there is no definitive cure to date [1,2]. The articular cartilage, covering the articular surface in the joints and forming an osteochondral unit in conjunction with the underlying subchondral bone, allows for joint stability, elasticity of compression, and shock absorption. Adult hyaline cartilage is an aneural, avascular tissue that uniquely consists of articular chondrocytes (5% of the total tissue volume) embedded in a tight extracellular matrix essentially composed of proteoglycans and type-II collagen. In absence of vascularization, cartilage defects resulting from trauma or in osteoarthritis (OA) do not have the ability to reliably restore their full, original architecture with mechanical competence [1,2,3,4]. Even following surgical interventions based on bone-marrow-stimulating techniques that allow to give access to bone-marrow-derived chondroregenerative progenitor cells in sites of injury (microfracturing, abrasion athroplasty, pridie drilling), the resulting repair tissue is chiefly made of fibrocartilage with type-I collagen as the main component and unable to withstand mechanical loads [1,2,3,4,5,6,7].

Cartilage gene therapy that aims at activating the intrinsic chondroreparative activities of bone-marrow-derived mesenchymal stem cells (MSCs) [8,9,10] may be envisaged by directly providing therapeutic gene vehicles in sites of cartilage injury [11,12] especially using clinically relevant recombinant adeno-associated virus (rAAV) vectors [13] that support the safe, effective, and durable transduction of these cells (up to 100% efficiencies for at least 3 weeks) without alteration of their chondrogenic potential [14,15,16,17,18,19,20]. Such a strategy has been reported using a number of therapeutic (chondrogenic) candidates such as the transforming growth factor beta (TGF-β) [14,18], basic fibroblast growth factor (FGF-2) [16], insulin-like growth factor I (IGF-I) [19], and the sex-determining region Y-type high-mobility group box 9 transcription factor (SOX9) alone [17] or combined with TGF-β [20], thus promoting the chondrogenic differentiation of the cells only to a certain extent and showing the need to explore new setups and combinations that might be more valuable than single treatments [12,21].

Here, we therefore investigated the feasibility of co-overexpressing the pleiotropic and highly chondrogenic TGF-β and IGF-I genes in hMSCs via rAAV-mediated gene transfer in order to stimulate the reparative activities in these cells in vitro based on our previous findings showing the efficacy of each of the therapeutic constructs in hMSCs independently [18,19]. Our results indicate that successful, prolonged concomitant overexpression of TGF-β with IGF-I via rAAV led to enhanced proliferative, anabolic, and chondrogenic activities in hMSCs aggregate cultures relative to independent gene application (TGF-β with *lacZ* or IGF-I with *lacZ*) and control (reporter *lacZ*) treatment while restraining undesirable osteogenic and hypertrophic differentiation. These data provide a basis to locally treat and improve cartilage repair by directly co-delivering multiple therapeutic rAAV vectors in sites of cartilage injury.

## 2. Results

### 2.1. Successful rAAV-Mediated TGF-β and IGF-I Co-Overexpression in hMSC Aggregates

**Figure 1:**
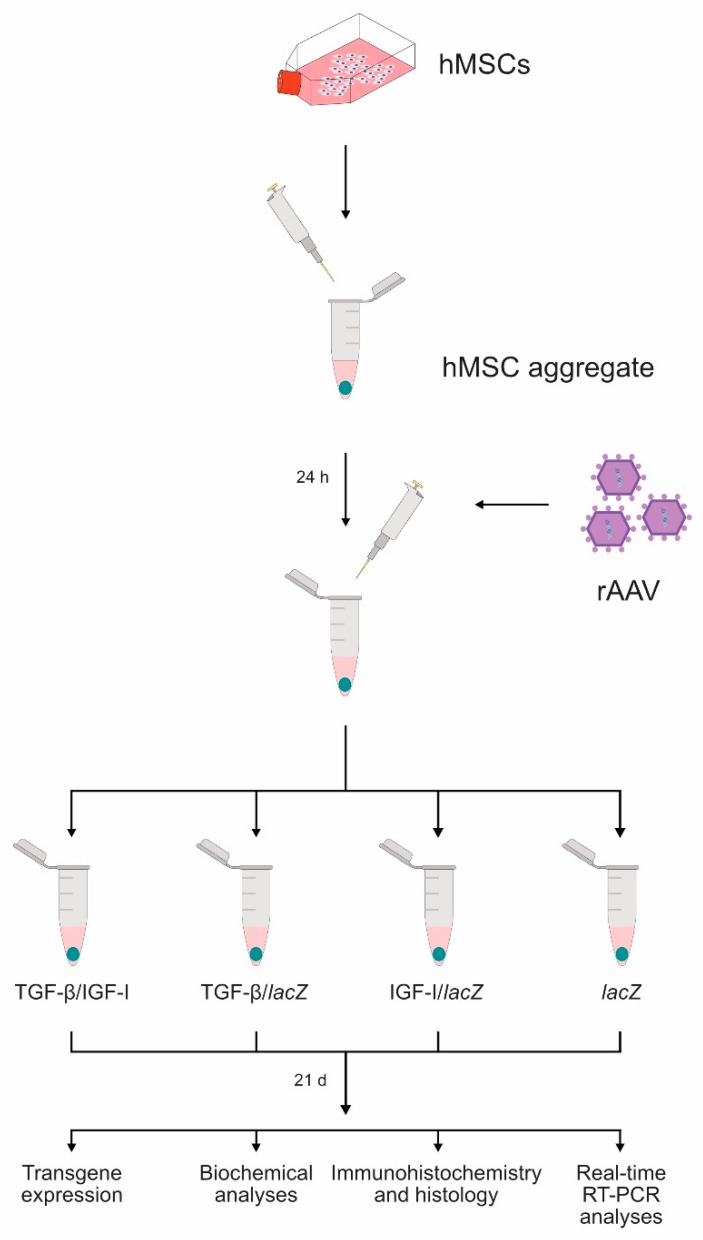
Study design. Human bone-marrow-derived mesenchymal stem cells (MSCs) were prepared in high-density aggregate cultures (2 × 10^5^ cells/aggregate) and placed in defined chondrogenic medium overnight prior to direct transduction with the recombinant adeno-associated virus (rAAV) vectors (TGF-β/IGF-I, TGF-β/*lacZ*, IGF-I/*lacZ*: 40 µL each vector/aggregate; *lacZ*: 80 µL vector/aggregate). rAAV-transduced aggregates were maintained in the defined chondrogenic medium for 21 days and analyzed to detect transgene expression and for biochemical analyses, immunohistochemistry and histology, and real-time RT-PCR analyses.

**Figure 2:**
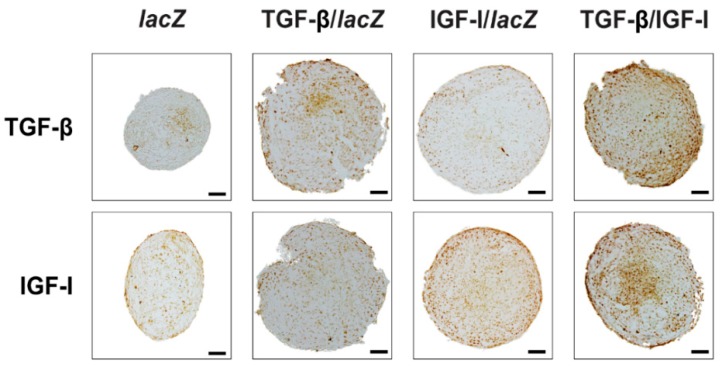
Detection of transgene (TGF-β, IGF-I) overexpression in rAAV-transduced hMSC aggregates. Aggregates were co-transduced with rAAV TGF-β/IGF-I, TGF-β/*lacZ*, IGF-I/*lacZ* or transduced with rAAV *lacZ* as described in Figure 1 and in the Materials and Methods. Samples were processed after 21 days to detect the expression of TGF-β and of IGF-I by immunohistochemistry (magnification ×10; all representative data). Scale bars: 100 µm.

hMSC aggregates were first transduced over time with rAAV according to the study design (Figure 1) in order to evaluate the ability of the vectors to promote the co-overexpression of the candidate TGF-β and IGF-I genes (TGF-β/IGF-I) relative to independent gene application (TGF-β/*lacZ* or IGF-I/*lacZ*) and control (*lacZ*) treatment. Successful, durable rAAV-mediated expression of the TGF-β and IGF-I transgenes was achieved in the specific conditions examined, especially in the TGF-β/IGF-I aggregates (Table 1 and Table 2 and Figure 2).

The TGF-β production levels in the TGF-β/IGF-I aggregates were 1.8-, 2.7-, 6.4-, and 8.7-fold higher than in the *lacZ* aggregates on days 2, 7, 14, and 21, respectively (*p* ≤ 0.050) while they were 1.5-, 1.4-, 1.5-, and 1.7-fold higher than in the TGF-β/*lacZ* aggregates at similar time points (*p* = 0.030, *p* = 0.059, *p* = 0.070, and *p* = 0.056, respectively) and 1.7-, 2.3-, 5.5-, and 6.4-fold higher than in the IGF-I/*lacZ* aggregates (*p* ≤ 0.034) (Table 1). They were also higher in the TGF-β/*lacZ* versus *lacZ* (1.2-, 1.9-, 4.2, and 5-fold difference on days 2, 7, 14, and 21, *p* = 0.148, *p* = 0.040, *p* = 0.053, and *p* = 0.040, respectively) and IGF-I/*lacZ* aggregates at similar time points (1.2-, 1.6-, 3.6-, and 3.7- fold difference, *p* = 0.042, *p* = 0.015, *p* = 0.054, and *p* = 0.059, respectively). No or little difference instead was seen between the IGF-I/*lacZ* and *lacZ* aggregates at similar time points (*p* = 0.440, *p* = 0.087, *p* = 0.217, and *p* = 0.010, respectively).

The IGF-I production levels in the TGF-β/IGF-I aggregates were 1.8-, 1.5-, 1.4-, and 1.3-fold higher than in the *lacZ* aggregates on days 2, 7, 14, and 21 (*p* ≤ 0.030) while they were 1.2-, 1.2-, 1.1-, and 1.1-fold higher than in the IGF-I/*lacZ* aggregates at similar time points (*p* ≥ 0.120) and 1.6-, 1.4-, 1.4-, and 1.4-fold higher than in the TGF-β/*lacZ* aggregates (*p* = 0.052, *p* = 0.030, *p* = 0.014, and *p* = 0.003, respectively) (Table 1). They were also higher in the IGF-I/*lacZ* versus *lacZ* (1.5-, 1.3-, 1.3, and 1.3-fold difference on days 2, 7, 14, and 21, respectively, *p* ≤ 0.010) and TGF-β/*lacZ* aggregates (1.3-, 1.2-, 1.3-, and 1.4-fold difference, *p* ≤ 0.009). No difference was seen between the TGF-β/*lacZ* and *lacZ* aggregates at similar time points (*p* ≥ 0.100).

Overall, these results were corroborated by an evaluation of transgene expression over time (21 days) on histological stions from aggregates by immunohistochemistry (Figure 2 and Table 2). Immunoreactivity to TGF-β was more intense in the TGF-β/IGF-I versus *lacZ*, TGF-β/*lacZ*, and IGF-I/*lacZ* aggregates (3.4-, 1.1-, and 3.1-fold difference, respectively, *p* ≤ 0.030). It was also higher in the TGF-β/*lacZ* versus *lacZ* and IGF-I/*lacZ* aggregates (3.2- and 2.9-fold difference, *p* ≤ 0.020) while no difference was seen between the IGF-I/*lacZ* and *lacZ* aggregates (*p* = 0.500). Immunoreactivity to IGF-I was more intense in the TGF-β/IGF-I versus *lacZ*, TGF-β/*lacZ*, and IGF-I/*lacZ* aggregates (3.3-, 2.7-, and 1.1-fold difference, respectively, *p* ≤ 0.050). It was also higher in the IGF-I/*lacZ* versus *lacZ* and TGF-β/*lacZ* aggregates (3- and 2.5-fold difference, respectively, *p* ≤ 0.050) while no difference was seen between the TGF-β/*lacZ* and *lacZ* aggregates (*p* ≥ 0.050).

### 2.2. Biological and Chondrogenic Differentiation Activities of rAAV-Mediated TGF-β and IGF-I Co-Overexpression in hMSC Aggregates

hMSC aggregates were next transduced with rAAV in order to evaluate the ability of the TGF-β/IGF-I vector combination to stimulate the proliferative, metabolic, and chondrogenic differentiation activities of the cells over time (21 days) relative to independent gene application (TGF-β/*lacZ* or IGF-I/*lacZ*) and control (*lacZ*) treatment.

**Figure 3:**
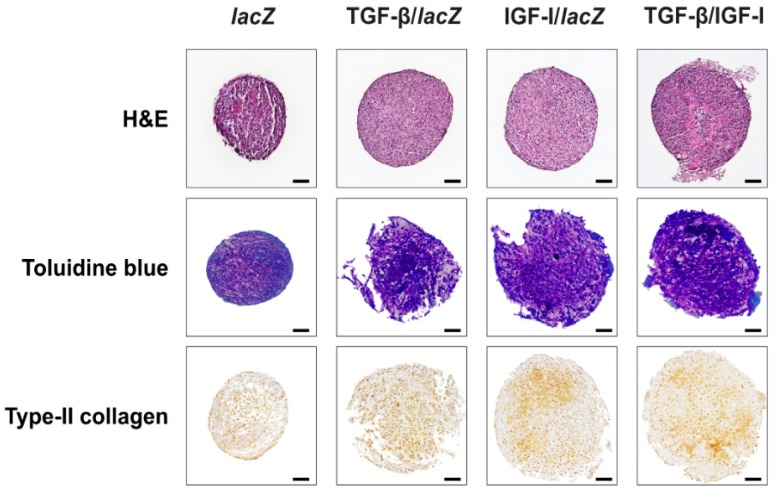
Biological and chondrogenic differentiation activities in rAAV-transduced hMSC aggregates. Aggregates were co-transduced with rAAV TGF-β/IGF-I, TGF-β/*lacZ*, IGF-I/*lacZ* or transduced with rAAV *lacZ* as described in Figure 1 and Figure 2 and in the Materials and Methods. Samples were processed after 21 days to evaluate cellularity (H and E staining) and the deposition of matrix proteoglycans (toluidine blue staining) and of type-II collagen (immunohistochemistry) (magnification ×10; all representative data). Scale bars: 100 µm.

The levels of cell proliferation measured by the DNA contents in the aggregates were higher in the TGF-β/IGF-I versus *lacZ*, TGF-β/*lacZ*, and IGF-I/*lacZ* aggregates (1.6-, 1.1-, and 1.4-fold difference, *p* = 0.004, *p* = 0.323, and *p* = 0.010, respectively) (Table 3). They were also higher in the TGF-β/*lacZ* versus *lacZ* and IGF-I/*lacZ* aggregates (1.4- and 1.2-fold difference, *p* = 0.073 and *p* = 0.094, respectively) and in the IGF-I/*lacZ* versus *lacZ* aggregates (1.2-fold difference, *p* = 0.010). Overall, these findings were substantiated by an estimation of the cell densities on H and E-stained histological stions from aggregates (Figure 3 and Table 2) revealing higher densities in the TGF-β/IGF-I versus *lacZ*, TGF-β/*lacZ*, and IGF-I/*lacZ* aggregates (3-, 1.6-, and 2.5-fold difference, respectively, *p* ≤ 0.010), in the TGF-β/*lacZ* versus *lacZ* and IGF-I/*lacZ* aggregates (1.9- and 1.6-fold difference, respectively, *p* ≤ 0.010), and in the IGF-I/*lacZ* versus *lacZ* aggregates (1.2-fold difference, *p* = 0.106).

The chondrogenic differentiation and matrix synthetic activities assessed by monitoring the proteoglycan contents and the intensities of toluidine blue staining and of type-II collagen immunostaining were higher in the TGF-β/IGF-I versus *lacZ*, TGF-β/*lacZ*, and IGF-I/*lacZ* aggregates (proteoglycans: 3.3-, 1.3-, and 1.7-fold difference, respectively, *p* ≤ 0.030; toluidine blue: 1.6-, 1.1-, and 1.2-fold difference, *p* = 0.007, *p* = 0.196, and *p* = 0.030, respectively; type-II collagen: 2.9-, 1.3-, and 1.7-fold difference, respectively, *p* ≤ 0.013) (Table 2 and Table 3 and Figure 3). They were also higher in the TGF-β/*lacZ* versus *lacZ* and IGF-I/*lacZ* aggregates (proteoglycans: 2.7- and 1.3-fold difference respectively, *p* ≥ 0.050; toluidine blue: 1.5- and 1.2-fold difference, *p* = 0.008 and *p* = 0.091; type-II collagen: 2.3- and 1.3-fold difference, *p* ≤ 0.024) and in the IGF-I/*lacZ* versus *lacZ* aggregates (proteoglycans: 2-fold difference, *p* ≥ 0.050; toluidine blue: 1.3-fold difference, *p* = 0.108; type-II collagen: 1.7-fold difference, *p* ≤ 0.001).

### 2.3. Effects of rAAV-Mediated TGF-β and IGF-I Co-Overexpression Upon the Osteogenic and Hypertrophic Differentiation Processes in hMSC Aggregates

hMSC aggregates were then transduced with rAAV in order to evaluate the capacity of the TGF-β/IGF-I vector combination to restrain the osteogenic and hypertrophic differentiation activities of the cells over time (21 days) relative to independent gene application (TGF-β/*lacZ* or IGF-I/*lacZ*) and control (*lacZ*) treatment.

**Figure 4:**
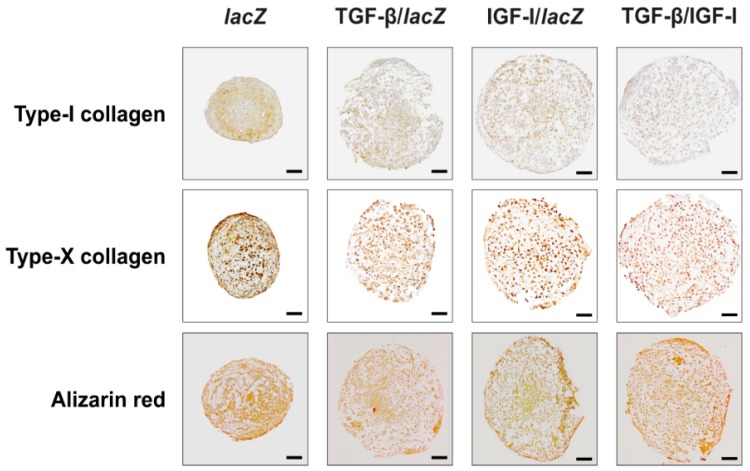
Osteogenic and hypertrophic differentiation processes in rAAV-transduced hMSC aggregates. Aggregates were co-transduced with rAAV TGF-β/IGF-I, TGF-β/*lacZ*, IGF-I/*lacZ* or transduced with rAAV *lacZ* as described in Figure 1, Figure 2 and Figure 3 and in the Materials and Methods. Samples were processed after 21 days to evaluate the deposition of type-I and -X collagen (immunohistochemistry) and matrix mineralization (alizarin red staining) (magnification ×10; all representative data). Scale bars: 100 µm.

The osteogenic differentiation activities estimated by monitoring the intensities of type-I collagen immunostaining were lower in the TGF-β/IGF-I versus *lacZ*, TGF-β/*lacZ*, and IGF-I/*lacZ* aggregates (2-, 1.2-, and 1.5-fold difference, *p* ≤ 0.001, *p* = 0.308, and *p* = 0.048, respectively) (Figure 4 and Table 2). They were also lower in the TGF-β/*lacZ* versus *lacZ* and IGF-I/*lacZ* aggregates (1.7- and 1.3-fold difference, *p* = 0.005 and *p* = 0.112, respectively) and in the IGF-I/*lacZ* versus *lacZ* aggregates (1.3-fold difference, *p* = 0.005).

The hypertrophic differentiation activities analyzed by evaluating the intensities of type-X collagen immunostaining and those of alizarin red staining were lower in the TGF-β/IGF-I versus *lacZ*, TGF-β/*lacZ*, and IGF-I/*lacZ* aggregates (type-X collagen: 2.6-, 1.7-, and 2.2-fold difference, respectively, *p* ≤ 0.009; alizarin red: 2.6-, 1.5-, and 2.2-fold difference, respectively, *p* ≤ 0.020) (Figure 4 and Table 2). They were also lower in the TGF-β/*lacZ* versus *lacZ* and IGF-I/*lacZ* aggregates (type-X collagen: 1.5- and 1.3-fold difference, *p* ≤ 0.010; alizarin red: 1.8- and 1.5-fold difference, *p* ≤ 0.040) and in the IGF-I/*lacZ* versus *lacZ* aggregates (type-X collagen: 1.2-fold difference, *p* = 0.004; alizarin red: 1.2-fold difference, *p* = 0.052).

### 2.4. Real-Time RT-PCR Analyses in hMSC Aggregates Following rAAV-Mediated TGF-β and IGF-I Co-Overexpression

**Figure 5:**
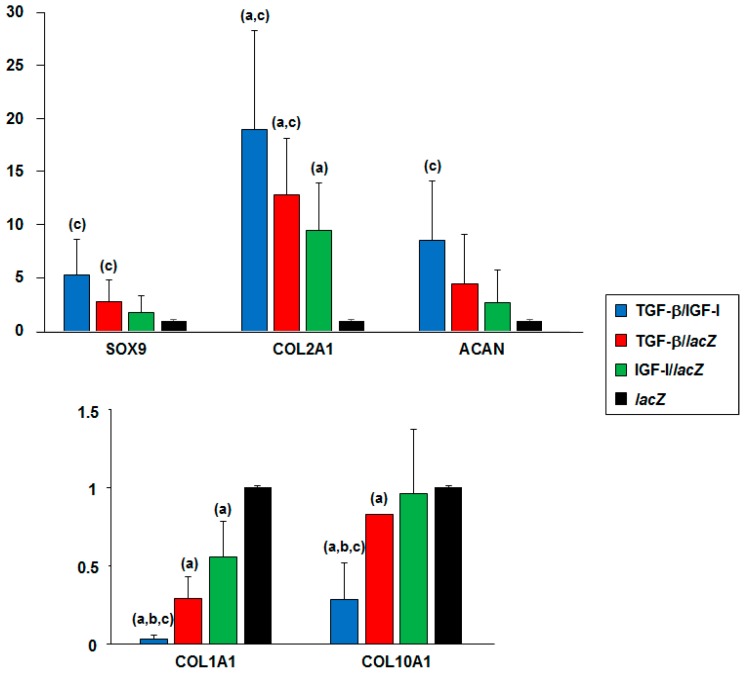
Real-time RT-PCR analyses in rAAV-transduced hMSC aggregates. aggregates were co-transduced with rAAV TGF-β/IGF-I, TGF-β/*lacZ*, IGF-I/*lacZ* or transduced with rAAV *lacZ* as described in Figure 1, Figure 2, Figure 3 and Figure 4 and in the Materials and Methods. Samples were processed after 21 days to monitor the gene expression profiles by real-time RT-PCR as described in the Materials and Methods. The genes analyzed included the transcription factor SOX9, type-II collagen (*COL2A1*), aggrecan (*ACAN*), type-I collagen (*COL1A1*), and type-X collagen (*COL10A1*), with GAPDH serving as a housekeeping gene and internal control. Threshold cycle (*Ct*) values were obtained for each target and GAPDH as a control for normalization, and fold inductions (relative to *lacZ* aggregates) were measured using the 2^−∆∆Ct^ method. Statistically significant relative to ^a^ *lacZ*, ^b^ TGF-β/*lacZ*, and ^c^ IGF-I/*lacZ*.

Overall, these findings were confirmed by the results of a real-time RT-PCR analysis performed in hMSC aggregates transduced over time (21 days) with the TGF-β/IGF-I vector combination relative to independent gene application (TGF-β/*lacZ* or IGF-I/*lacZ*) and control (*lacZ*) treatment (Figure 5).

Enhanced chondrogenic differentiation was evidenced by increased SOX9 expression in the TGF-β/IGF-I versus *lacZ*, TGF-β/*lacZ*, and IGF-I/*lacZ* aggregates (5.3-, 1.9-, and 2.9-fold difference, *p* = 0.083, *p* = 0.059, and *p* = 0.047, respectively), in the TGF-β/*lacZ* versus *lacZ* and IGF-I/*lacZ* aggregates (2.7- and 1.5-fold difference, *p* = 0.146 and *p* = 0.048, respectively), and in the IGF-I/*lacZ* versus *lacZ* aggregates (1.8-fold difference, *p* = 0.240). Higher chondrogenic COL2A1 expression was also noted in the TGF-β/IGF-I versus *lacZ*, TGF-β/*lacZ*, and IGF-I/*lacZ* aggregates (19-, 1.5-, and 2-fold difference, *p* = 0.039, *p* = 0.058, and *p* = 0.039, respectively), in the TGF-β/*lacZ* versus *lacZ* and IGF-I/*lacZ* aggregates (12.8- and 1.4-fold difference, *p* ≤ 0.031), and in the IGF-I/*lacZ* versus *lacZ* aggregates (9.5-fold difference, respectively, *p* = 0.040). Increased chondrogenic ACAN expression was also observed in the TGF-β/IGF-I versus *lacZ*, TGF-β/*lacZ*, and IGF-I/*lacZ* aggregates (8.6-, 1.9-, and 3.2-fold difference, *p* = 0.072, *p* = 0.056, and *p* = 0.041, respectively), in the TGF-β/*lacZ* versus *lacZ* and IGF-I/*lacZ* aggregates (4.4- and 1.6-fold difference, *p* ≥ 0.102), and in the IGF-I/*lacZ* versus *lacZ* aggregates (2.7-fold difference, *p* = 0.223).

Reduced osteogenic differentiation was observed by decreased COL1A1 expression in the TGF-β/IGF-I versus *lacZ*, TGF-β/*lacZ*, and IGF-I/*lacZ* aggregates (30.4-, 8.7-, and 16.9-fold difference, respectively, *p* ≤ 0.032), in the TGF-β/*lacZ* versus *lacZ* and IGF-I/*lacZ* aggregates (3.5- and 1.9-fold difference, *p* = 0.006 and *p* = 0.146, respectively), and in the IGF-I/*lacZ* versus *lacZ* aggregates (1.8-fold difference, *p* = 0.039).

Hypertrophic differentiation (COL10A1 expression) was also restrained in the TGF-β/IGF-I versus *lacZ*, TGF-β/*lacZ*, and IGF-I/*lacZ* aggregates (3.6-, 2.9-, and 3.4-fold difference, *p* = 0.017, *p* = 0.032, and *p* = 0.036, respectively), in the TGF-β/*lacZ* versus *lacZ* and IGF-I/*lacZ* aggregates (1.2-fold difference, *p* ≤ 0.001 and *p* = 0.063, respectively), and in the IGF-I/*lacZ* versus *lacZ* aggregates (1.1-fold difference, *p* = 0.440).

## 3. Discussion

Direct application of therapeutic gene transfer vectors such as highly effective, clinically adapted rAAV constructs [13] to articular cartilage lesions is a promising strategy to stimulate the chondroreparative activities of the bone-marrow-derived MSCs that repopulate the defects in vivo [11,12,23]. While a number of candidate sequences have been reported for their efficacy to achieve this goal (TGF-β, FGF-2, IGF-I, SOX9) [14,16,17,18,19,20], none were able to accurately promote chondrogenic cell differentiation, showing the critical need to explore new lines of research. Based on the perception that combined treatments may be more beneficial than single approaches [12,21], we tested the hypothesis that concomitant administration of two highly chondrogenic genes (TGF-β and IGF-I) stimulates the pro-chondrogenic activities of human MSCs relative to single and control gene conditions.

Our results first demonstrate that simultaneous expression of TGF-β and IGF-I was significantly achieved in hMSCs via rAAV over extended periods of time (21 days) and relative to independent candidate gene delivery and to reporter gene treatment, concordant with previous findings using rAAV [14,16,17,18,19,20], probably due to the good maintenance of the constructs in such cells without vector interference [20]. Interestingly, the production levels of each of the candidate genes were always more elevated in TGF-β/IGF-I co-transduced cells than in those treated with each individual gene, reflecting a possible interactive regulation of growth factor expression as observed in chondrocytes [24]. The levels of growth factor production in hMSCs modified with TGF-β/*lacZ* or by IGF-I/*lacZ* were in the range of those reported in our previous studies when using only TGF-β or IGF-I at comparable MOIs (up to ~ 70–100 pg/mL at MOI = 8) [18,19].

The data next indicate that durable, effective co-overexpression of TGF-β and IGF-I activated the proliferative, biosynthetic, and chondrogenic activities over time in hMSCs to levels that were more important than when using independent candidate and reporter gene treatments, as a probable result of growth factor synergy [25]. This is in good agreement with the properties of these growth factors [8,9,10,14,25] and with our previous work using rAAV-hTGF-β and rAAV-hIGF-I separately [18,19]. Most notably, combined rAAV-mediated TGF-β and IGF-I gene transfer delayed osteogenic and hypertrophic differentiation in hMSCs relative to independent candidate gene and reporter gene conditions, possibly due to enhanced levels of anti-hypertrophic SOX9 expression achieved in TGF-β/IGF-I cells [17,26,27]. Similar trends were also noted upon single TGF-β/*lacZ* and IGF-I/*lacZ* treatments versus *lacZ* which is in contrast with our previous findings where each vector (and corresponding growth factor expression) triggered osteogenic and hypertrophic activities in hMSCs [18,19]. However, in these reports, a higher MOI was applied to the cells (MOI = 20 versus MOI = 8 in the present study, i.e., a 2.5-fold difference), suggesting that the careful choice of vector doses will be critical for optimal therapy.

In conclusion, our data uncover the value of combined rAAV-mediated overexpression of TGF-β and IGF-I to stimulate the chondrogenesis of hMSCs in vitro, as a future, direct therapeutic tool for administration in sites of cartilage damage. Work is currently ongoing to confirm the findings in a human osteochondral defect model via implantation of such co-modified hMSCs [28] and in animal MSCs and in a translational (orthotopic) animal model of focal cartilage defects [29,30]. These evaluations provide a basis for improved cartilage repair upon application of using multiple candidate rAAV vectors in vivo.

## 4. Materials and Methods

### 4.1. Study Design

High-density aggregate cultures of human bone-marrow-derived MSCs were prepared in defined chondrogenic medium and then transduced with the different rAAV vectors (total MOI = 8) using the candidate rAAV-hTGF-β/rAAV-hIGF-I combination (TGF-β/IGF-I; 40 µL + 40 µL vectors/aggregate) relative to the control conditions of independent vectors rAAV-hTGF-β/rAAV-*lacZ* (TGF-β/*lacZ*: 40 µL + 40 µL vectors/aggregate) or rAAV-hIGF-I/rAAV-*lacZ* (IGF-I/*lacZ*: 40 µL + 40 µL vectors/aggregate) and to the lack of therapeutic gene (rAAV-*lacZ* alone, i.e., *lacZ*: 80 µL vector/aggregate). A 1:1 ratio of vector combinations was selected based on our previous work to ensure strict comparison [20]. Aggregates were kept in defined chondrogenic medium for 21 days and processed to weekly monitor transgene expression [16,18,19,20] and to perform biochemical analyses, immunohistochemistry and histology, and real-time RT-PCR analyses at the time point of 21 days where hMSC chondrodifferentiation fully occurs [8,9,10] (Figure 1).

### 4.2. Reagents

All reagents were from Sigma, Munich (Germany) unless otherwise indicated. The dimethylmethylene blue dye (DMMB) was from Serva (Heidelberg, Germany). The anti- TGF-β (V) antibody was from Santa Cruz Biotechnology (Heidelberg, Germany), the anti-IGF-I (AF-291-NA) from R&D Systems (Wiesbaden, Germany), the anti-type-II collagen (II-II6B3) antibody from NIH Hybridome Bank (University of Iowa, Ames, IA, USA), the anti-type-I collagen (AF-5610) antibody from Acris Antibodies (Hiddenhausen, Germany), and the anti-type-X collagen (COL10) antibody from Sigma. Biotinylated sondary antibodies and ABC reagent were from Vector Laboratories (Alexis Deutschland, Grünberg, Germany). The TGF-β and IGF-I Quantikine ELISAs were from R&D Systems. The AAVanced Concentration Reagent was from System Bioscience (Heidelberg, Germany).

### 4.3. Cell Culture

Bone marrow aspirates were obtained from distal femurs of patients undergoing total knee arthroplasty (*n* = 3). All patients included in this study declared in advance informed consent. All proceedings and methods were executed in compliance with the Helsinki Declaration. The study was approved by the Ethics Committee of the Saarland Physicians Council. Bone-marrow-derived hMSCs were first isolated according to standard protocols [16,17,18,19,20,22] by washing and centrifuging the aspirates (about 15 mL per each patient) in Dulbecco’s modified Eagle’s Medium (DMEM). The pellet maintained was resuspended in red blood cell lysing buffer and DMEM in equal ratios. The obtained mixture was washed, pelleted, and resuspended in DMEM containing 10% fetal bovine serum with 100 U/mL penicillin and 100 µL/mL streptomycin. Cells were plated in T75 flasks and kept in an incubator at 37 °C with 5% CO_2_ overnight. The medium was next removed and substituted by growth medium containing recombinant FGF-2 (1 ng/mL). Medium was exchanged every 2–3 days. Proliferating cells were replated when reaching a density of 85%. The hMSCs were used at passage 1–2.

### 4.4. Plasmids and rAAV Vectors

All rAAV vectors were derived from the AAV-2-based vector plasmid pSSV9 [31,32]. rAAV-*lacZ* carries the *lacZ* gene coding for the *Escherichia coli* β-galactosidase (β-gal), rAAV-hTGF-β a human transforming growth factor beta 1 (hTGF-β) complementary DNA (cDNA) sequence, and rAAV-hIGF-I a human insulin-like growth factor I (hIGF-I) cDNA sequence, all controlled by the cytomegalovirus immediate-early (CMV-IE) promoter/enhancer [16,17,18,19,20]. rAAV were packaged as conventional (not self-complementary) vectors using a helper-based system maintaining Adenovirus 5 and pAd8 for helper functions in the 293 cell line (an adenovirus transformed human embryonic kidney packaging cell line) [16,17,19,20]. The vector preparations were purified by dialysis using the AAVanced Concentration Reagent and titrated by real-time PCR as previously described (approximately 10^10^ transgene copies/mL and ratio recombinant functional viral particles-to-recombinant viral particles of about 1/500) [16,17,18,19,20].

### 4.5. rAAV-Mediated Gene Transfer

hMSCs were pelleted (2 × 10^5^ cells/aggregate) and kept in 150 µL chondrogenic medium (DMEM high glucose 4.5 g/L, ITS^+^ Premix, 1 mM pyruvate, ascorbate 2-phosphate 37.5 ng/mL, 10^−7^ M dexamethasone, TGF-β3 10 ng/mL) overnight before transduction [16,17,18,19,20]. The hMSC aggregates were then transduced with the rAAV vector conditions: TGF-β/IGF-I, TGF-β/*lacZ*, IGF-I/*lacZ* (40 µL each vector/aggregate) and *lacZ* as control condition (80 µL vector/aggregate). rAAV vectors (total MOI = 8) were directly applied to the aggregates, left at room temperature for one min, filled up with 150 µL chondrogenic medium and kept at 37 °C under 5% CO_2_ for 24 h. The supernatant was then discarded and the pellets kept as high-density aggregate cultures in chondrogenic medium for up to 21 days. The chondrogenic medium was exchanged every 2–3 days.

### 4.6. Transgene Expression

Transgene expression (TGF-β and IGF-I) was quantitatively estimated by respective ELISAs. Briefly, aggregates were washed with serum-free medium at the denoted time points and kept in 150 µL DMEM for 24 h. Cell supernatants were then collected and centrifuged to remove debris and processed according to the manufacturer’s recommendations for evaluation using a GENios spectrophotometer/fluorometer (Tecan, Crailsheim, Germany) [18,19,20]. Transgene expression was also determined by immunohistochemical analyses using paraffin-embedded stions of the constructs and specific antibodies, biotinylated sondary antibodies, and the ABC detection method using diaminobenzidine (DAB) as the chromogen [16,17,18,19,20]. Samples were evaluated under light microscopy (Olympus BX 45, Hamburg, Germany).

### 4.7. Histological and Immunohistochemical Analyses

Cell aggregates were harvested, fixed in formaldehyde (4%), and dehydrated in graded alcohol [16,17,18,19,20]. Histological and immunohistochemical analyses were performed on paraffin-embedded sections of the constructs (5 µm). Sections were stained with hematoxylin and eosin (H and E) (cellularity), toluidine blue (matrix proteoglycans), and alizarin red (matrix mineralization) [16,17,18,19,20]. Immunohistochemical analyses were performed to detect type-II, -I, and -X collagen expression using specific primary antibodies, biotinylated secondary antibodies, and the ABC detection method using DAB as the chromogen [16,17,18,19,20]. Samples were examined under light microscopy (Olympus BX 45).

### 4.8. Histomorphometric Analyses

The transduction efficiencies (ratio cells positive for TGF-β or IGF-I immunoreactivity to the total cell number), cell densities (cells/mm^2^), and the intensities of toluidine blue and alizarin red staining and those of type-II, -I, and -X collagen immunostaining (ratio tissue surface showing positive immunoreactivity for particular collagen to the total tissue surface) were measured at three random sites standardized for their surface or using serial histological or immunohistochemical stions using the SIS analysisSIS program (Olympus), Adobe photoshop (Adobe Systems, Unterschleissheim, Germany), and Scion Image (Scion Corporation, Frederick, MD, USA) [16,17,18,19,20]. Stained stions were scored for uniformity and intensity according to a modified Bern Score grading system [22] as: 0 (no staining), 1 (heterogenous and/or weak staining), 2 (homogeneous and/or moderate staining), 3 (homogeneous and/or intense staining), and 4 (very intense staining).

### 4.9. Biochemical Analyses

Aggregates were harvested and digested in papain to monitor the DNA contents by Hoechst 33258 fluorometric assay, the proteoglycan contents by binding to dimethylmethylene blue dye (DMMB), and the total cellular protein contents for normalization by using a protein assay (Pierce Thermo Scientific, Fisher Scientific, Schwerte, Germany) [16,17,18,19,20]. All measurements were performed using a GENios spectrophotometer/fluorometer (Tecan).

### 4.10. Real-time RT-PCR Analyses

Total RNA was extracted from aggregates (RNeasy Protect Mini kit, Qiagen, Hilden, Germany) for reverse transcription (1st Strand cDNA Synthesis kit, avian myeloblastis virus—AMV) (Roche Applied Science, Mannheim, Germany). cDNA amplification was performed via SYBR Green real-time RT-PCR (Stratagene, Agilent Technologies, Waldbronn, Germany) maintaining initial incubation (95 °C,10 min), 55 cycles of amplification (denaturation (95 °C, 30 s), annealing (55 °C, 1 min), extension (72 °C, 30 s), denaturation (95 °C, 1 min), and final incubation (55 °C, 30 s) [16,17,18,19,20]. The following primers were used: SOX9 (chondrogenic marker) (forward 5′-ACACACAGCTCACTCGACCTTG-3′; reverse 5′-GGGAATTCTGGTTGGTCCTCT-3′), type-II collagen (COL2A1, chondrogenic marker) (forward 5′-GGACTTTTCTCCCCTCTCT-3; reverse 5′-GACCCGAAGGTCTTACAGGA-3′), aggrecan (ACAN, chondrogenic marker) (forward 5′-GAGATGGAGGGTGAGGTC-3′; reverse 5′-ACGCTGCCTCGGGCTTC-3′), type-I collagen (COL1A1, osteogenic marker) (forward 5′-ACGTCCTGGTGAAGTTGGTC-3′; reverse 5′-ACCAGGGAAGCCTCTCTCTC-3′), type-X collagen (COL10A1, hypertrophic marker) (forward 5′-CCCTCTTGTTAGTGCCAACC-3′; reverse 5′-AGATTCCAGTCCTTGGGTCA-3′), and glyceraldehyde-3-phosphate dehydrogenase (GAPDH) (housekeeping gene and internal control) (forward 5′-GAAGGTGAAGGTCGGAGTC-3′; reverse 5′-GAAGATGGTGATGGGATTTC-3′) [16,17,18,19,20]. Control conditions included reactions using water and nonreverse-transcribed mRNA. Specificity of the products was confirmed by melting curve analysis and agarose gel electrophoresis. The threshold cycle (Ct) value for each gene of interest was measured for each amplified sample using MxPro QPCR software (Stratagene), and values were normalized to GAPDH expression by using the 2^−∆∆Ct^ method [16,17,18,19,20].

### 4.11. Statistical Analysis

Each condition was performed in duplicate in three independent experiments for each patient. The values obtained are expressed as mean ± standard deviation (SD). A *t*-test was employed with *p* < 0.05 considered statistically significant.

## Figures and Tables

**Table 1:** Transgene expression in rAAV-transduced hMSC aggregates.

Assay		*lacZ*	TGF-β/*lacZ*	IGF-I/*lacZ*	TGF-β/IGF-I
**TGF-β**	day 2	162 ± 158	196 ± 121 ^c^	166 ± 127	286 ± 169 ^a,b,c^
	day 7	89 ± 93	169 ± 119 ^a,c^	105 ± 93	241 ± 183 ^a,c^
	day 14	19 ± 8	80 ± 73	22 ± 6	121 ± 97 ^a,c^
	day 21	11 ± 3	55 ± 51 ^a^	15 ± 4 ^a^	96 ± 80 ^a,c^
**IGF-I**	day 2	52 ± 16	59 ± 22	76 ± 27 ^a,b^	93 ± 53 ^a^
	day 7	52 ± 11	53 ± 14	65 ± 17 ^a,b^	76 ± 29 ^a,b^
	day 14	54 ± 13	52 ± 15	69 ± 15 ^a,b^	73 ± 17 ^a,b^
	day 21	53 ± 16	49 ± 16	67 ± 18 ^a,b^	71 ± 16 ^a,b^

The levels of TGF-β and IGF-I production are in pg/mL. Data are given as mean ± SD. Statistically significant relative to ^a^ *lacZ*, ^b^ TGF-β/*lacZ*, and ^c^ IGF-I/*lacZ*.

**Table 2:** Histomorphometric analyses in rAAV-transduced hMSC aggregates (day 21).

Assay	*lacZ*	TGF-β/*lacZ*	IGF-I/*lacZ*	TGF-β/IGF-I
**TGF-β^+^ cells**	26 ± 6	84 ± 6 ^a,c^	29 ± 12	89 ± 4 ^a,b,c^
**IGF-I^+^ cells**	27 ± 14	33 ± 8	82 ± 4 ^a,b^	90 ± 5 ^a,b,c^
**Cell densities**	4817 ± 1348	9061 ± 840 ^a,c^	5628 ± 1116	14,267 ± 1224 ^a,b,c^
**Toluidine blue**	2.50 ± 0.58	3.75 ± 0.50 ^a^	3.25 ± 0.50	4.00 ± 0.01 ^a,c^
**Type-II collagen**	1.10 ± 0.32	2.50 ± 0.67 ^a,c^	1.92 ± 0.67 ^a^	3.20 ± 0.42 ^a,b,c^
**Type-I collagen**	2.60 ± 0.51	1.50 ± 0.52 ^a^	2.00 ± 0.67 ^a^	1.30 ± 0.89 ^a,c^
**Type-X collagen**	3.40 ± 0.67	2.20 ± 0.83 ^a,c^	2.90 ± 0.67 ^a^	1.30 ± 0.65 ^a,b,c^
**Alizarin red**	2.90 ± 0.64	1.60 ± 0.74 ^a,c^	2.40 ± 0.52	1.10 ± 0.64 ^a,b,c^

TGF-β^+^ and IGF-I^+^ cells are in %. The cell densities are as cells/mm^2^. Stained (toluidine blue, alizarin red) and immunostained (type-II, -I, and -X collagen) stions were scored for uniformity and intensity according to a modified Bern Score grading system [22] as: 0 (no staining), 1 (heterogenous and/or weak staining), 2 (homogeneous and/or moderate staining), 3 (homogeneous and/or intense staining), and 4 (very intense staining). Data are given as mean ± SD. Statistically significant relative to ^a^ *lacZ*, ^b^ TGF-β/*lacZ*, and ^c^ IGF-I/*lacZ*.

**Table 3:** Biological activities in rAAV-transduced hMSC aggregates (day 21).

Assay	*lacZ*	TGF-β/*lacZ*	IGF-I/*lacZ*	TGF-β/IGF-I
**DNA**	0.029 ± 0.006	0.042 ± 0.018 ^a^	0.034 ± 0.009 ^a^	0.046 ± 0.010 ^a,c^
**Proteoglycans**	0.3 ± 0.1	0.8 ± 0.7	0.6 ± 0.5	1.0 ± 0.8 ^a,b,c^

The DNA contents are in ng/mg total proteins and the proteoglycan contents in µg/mg total proteins. Data are given as mean ± SD. Statistically significant relative to ^a^ *lacZ*, ^b^ TGF-β/*lacZ*, and ^c^ IGF-I/*lacZ*.
